# Enhancing growth, antioxidant capacity, and immune response in tilapia (*Oreochromis niloticus*) through curcumin supplementation across varied stocking density paradigms

**DOI:** 10.1371/journal.pone.0311146

**Published:** 2024-11-20

**Authors:** Wajeeha Komal, Shafaq Fatima, Qandeel Minahal, Razia Liaqat

**Affiliations:** 1 Faculty of Natural Sciences, Department of Zoology, Lahore College for Women University, Lahore, Punjab, Pakistan; 2 Department of Biological Sciences, Purdue University Fort Wayne, Wayne, Indiana, United States of America; Cairo University, Faculty of Science, EGYPT

## Abstract

The present study evaluated the effects of curcumin on growth, immune and antioxidant response in tilapia (*Oreochromis niloticus*). An optimum dose of curcumin was investigated by feeding four different levels of this compound in combination with three different regimes of stocking density (12 treatments). Fish were reared at three densities; low density (LD = 1.50 kg/m^3^), medium density (MD = 3.00 kg/m^3^), and high density (HD = 4.50 kg/m^3^). Each treatment was fed with four different levels of dietary supplementation of curcumin (C0 = 0 mg/kg, C1 = 50 mg/kg, C2 = 100 mg/kg, and C3 = 150 mg/kg) for 60 days. Each treatment has three replicates (n = 50/replicate in LD, 100/replicate in MD, 150/ replicate in HD). Although better growth was observed in MD, however treatments at all densities fed with C1 diet showed improved growth as compared to other diets. Chemical composition of fish and activity of amylase, lipase and protease in all treatments were noted to be similar. Levels of antioxidant enzymes (catalase, superoxide dismutase and glutathione peroxidase) and cortisol in MD and HD treatments were similar to those in LD treatment. However, fish fed with C1 diet in each density treatment showed the lowest values of antioxidant enzymes. Similarly, the levels of malondialdehyde were noted to be similar in MD and HD treatments as compared to that in LD. Its levels were lower in fish fed with C1 and C3 diets in all density treatments. Expression of pro-opiomelanocortin-α (POMC-α), Somatostatins-1 (SST-1) and Interleukin 1-β (IL-1β) did not increase in MD and HD treatments in response to high stocking density when compared with LD treatment. The lowest levels of these genes were noted in fish fed with C2 and C3 diets in all treatments. In conclusion, supplementation of curcumin in diet of tilapia improved growth and antioxidant response in tilapia. optimum dose of curcumin for tilapia culture is 50 mg/kg at the density of 3.00 kg/m^3^which might be further investigated for intensive culture.

## 1 Introduction

Fish is widely recognized as a vital source of animal protein for human world widely [1]. There is a compelling demand to enhance aquaculture development to meet the rising global demand for human population. Global production of tilapia in year 2020 was seven million tonnes [2]. Nile tilapia (*Oreochromis niloticus*) stands out as a valuable species for aquaculture due to its rapid growth, minimal reliance on expensive animal protein in its diet and adaptability to high stocking densities in intensive production systems. The success and profitability of intensive fish culture systems depend on the growth rate of the fish and the stocking density employed [3–7].

High stocking density decrease the production by affects the physiology of fish by decreasing growth rate [8], increasing levels of cortisol and mortality and causing oxidative stress [9]. Elevating the number of fish in striking the space tends to harm their growth performance [10] as it deteriorates water quality, impacts social behavior, and uncontrolled metabolic rates due to the stress linked to crowding [11]. The fish stress responses are closely linked to hormonal reactions in the brain. The hypothalamic-pituitary interrenal (HPI) axis plays a key role in this process, triggering the production of corticotropin-releasing hormone (CRH) in the hypothalamus [12]. To assess how stocking density affects fish physiology, common parameters such as blood composition and alkaline phosphatase (ALP) levels are often examined [13]. Research indicates that high stocking density can have adverse effects on various blood parameters, including hematology and blood biochemistry [14] and can lead to chronic stress by increasing cortisol and glucose levels [15].

Crowding in production systems also causes oxidative stress, evident in the elevated production of reactive oxygen species (ROS) [16]. Accumulation of free radicals in the form of ROS occurs more rapidly leading to various forms of cellular damage i.e. mutation in nucleic acid [17]. Moreover, oxidative stress influences the expression of genes related to energy metabolism (peroxisome proliferator-activated receptor gamma coactivator 1-alpha (PGC-1α) [18], growth (Growth hormone, [19] Insulin-like growth factor-1 (IGF-1) [20], myostatin [21], immunological (nuclear factor-kβ) [22] and antioxidant enzymes (Nuclear factor erythroid 2-related factor 2 (Nrf2) [23], and other cellular defense proteins [22] in fishes. These biomolecules stimulate cascade of reactions to cope with damage caused by stress.

Fish experience changes in their innate and adaptive immune responses due to oxidative stress, starting with the activation of the HPA or HPI axis. This triggers the release of corticotropin-releasing factor (CRF), leading to the production of pituitary pro-opiomelanocortin (POMC) peptides, including adrenocorticotropic hormone (ACTH), which stimulates cortisol and other hormone synthesis to manage stress [24]. Somatostatins-1 (SST-1) are a diverse group of peptide hormones influencing growth, development and metabolism in vertebrates [25]. SST-1 which negatively affect growth hormone secretion, also play a role in regulating growth and metabolism [26]. Additionally, Interleukin 1-β (IL-1β), a proinflammatory cytokine, helps balance the immune system and alleviate stress by influencing the HPA axis [27].

This oxidative stress can be mitigated by supplementing with natural substances possessing antioxidant properties [28,29]. Recently researchers have found a positive connection between supplementing diets with antioxidants and reducing harmful effects such as health of fish and the activation of stress responses due to stocking density [30]. It also slows growth rates as in rainbow trout and grass carp (*Ctenopharyngodon idella*) juveniles [31,32], Nile tilapia (*Oreochromis niloticus*) [33,34], and largemouth bass (*Micropterus salmoides*) [35]. In this context, curcumin plays a role in repairing biomolecules, and membrane systems damaged by oxidative stress. in addition, it helps to maintain balance of normal physiological system and boosts the immune system [36]. Natural sources of antioxidants with higher phenolic compound levels have demonstrated the effectiveness in reducing oxidation, comparable to synthetic antioxidants [37–40]. When used as a preservative, curcumin can reduce oil oxidation and when included as a dietary supplement, it can enhance antioxidant capacity up to 80% [41]. The antioxidant effects of curcumin result from its ability to bind with free radicals, and provide a hydrogen atom [42].

Curcumin has the ability to decrease the generation of free radicals through the fenton reaction by binding to Fe^2+^/^3+^, Cu^2+^, [VO]^2+^ and Mn^2+^ [43]. It is crucial to acknowledge that curcumin is known to have low bioavailability (an average of 490 nmol/L in plasma) [44]. The antioxidant and cellular effects observed in vitro at high curcumin concentrations may not be replicated at physiological concentrations *in vivo* [40]. Additionally, *in vivo* curcumin is rapidly transformed into various metabolites through processes like glucuronidation and sulfation, either enzymatically or spontaneously [45]. Curcumin can also indirectly protect against free radicals by inhibiting reactive-oxygen-generating enzymes such as NADPH oxidase (NOX), lipoxygenase/cyclooxygenase, xanthine dehydrogenase/oxidase, and inducible nitric oxide synthase (iNOS) or by inducing antioxidant enzymes [46].

Present study conducted with different doses of curcumin against variant stocking density condition in tilapia. Study also determines the optimum dose of curcumin against different stocking density specifically at high stocking density. The objective of study was to assess the impact of a water-soluble curcumin formulation in the diet on the growth performance, stress physiology, antioxidant status, and gene expression of POMC-α, IL-1β and SST-1 in tilapia exposed to different stocking densities. This optimum dose could be used as dietary supplement in tilapia commercial culture to enhance growth and immune response at high stocking density.

## 2. Materials and methods

### 2.1. Diet preparation

In present study, commercial curcumin (C_21_H_20_O_6_: sigma aldrich, USA; purity ≥65%) was used as a feed supplement. Curcumin used in this study was sourced from *Curcuma longa* (Turmeric) powder with the formula weight of 368.38 g/mol. Its solubility color was from yellow to orange and with UV/VIS absorbance range of 420–430 nm. Treatment diets were prepared by mixing the finely ground ingredients with four levels of curcumin (C0 = 0 mg/kg, C1 = 50 mg/kg, C2 = 100 mg/kg, C3 = 150 mg/kg) and pellet of 1mm was prepared using mechanical pellet machine (Table 1). The pellets were air-dried at room temperature and stored at 4°C in sealed bags. Fish were fed at a proportion of 2% of their body mass on daily basis, twice a day. The recommended standard feeding ration for tilapia (*Oreochromis niloticus*) is 2% [47]. The feed utilized in this study contained 30% crude protein, aligning with the tilapia protein requirement range of 25–30%. The dietary requirement for tilapia in context to total energy content (calorie) is 2,500–3,000 kcal/kg and a fiber content ranging between 3% and 7% [48].

**Table 1:** Feed formulation with curcumin supplementation.

Ingredients (%)	C0	C1	C2	C3
Corn meal	28.00	28.00	28.00	28.00
Rice polish	12.00	12.00	12.00	12.00
Wheat bran	9.00	9.00	9.00	9.00
Canola meal	8.00	8.00	8.00	8.00
Soybean meal	36.00	36.00	36.00	36.00
Dicalcium phosphate	4.00	4.00	4.00	4.00
Methionine	0.79	0.79	0.79	0.79
Lysine	1.32	1.32	1.32	1.32
L-Threonine	0.89	0.89	0.89	0.89
Curcumin	0.00	5.00	10.00	15.00
Chemical composition of feed				
Moisture (%)	10.45	10.48	10.55	10.59
Crude protein (%)	30.00	30.00	30.00	30.00
Crude fat (%)	7.40	8.00	7.66	7.98
Crude ash (%)	7.98	7.11	7.09	7.16

### 2.2. Experimental design

**Fig 1:**
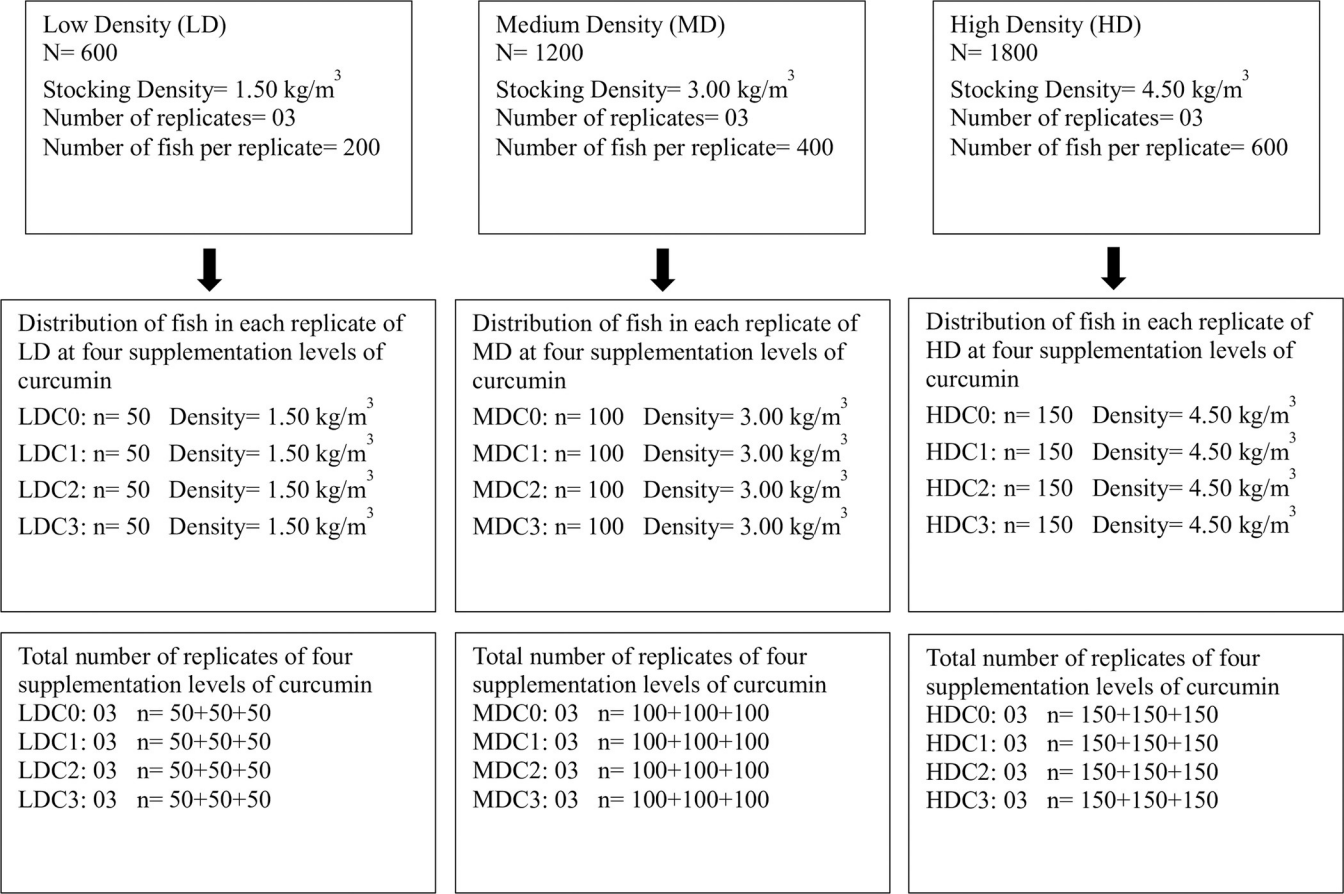
Distribution of fish in experimental design having three stocking densities (LD, MD, HD) and their replicates at four different level of curcumin supplementation (C0, C1, C2,C3).

Fish (n = 3600, initial weight = 30.00±1.20g) were procured from a local fish hatchery (Lahore, Pakistan) and transferred to Aquaculture facility, Lahore College for Women University. There was no mortality of fish during the transfer. This study was commenced after approval of Animal Ethics Committee of Department of Zoology, Lahore College for Women University (Approval #: Zoo/LCWU/932). Fish were randomly distributed in 36 fiber glass tanks (water volume/tank = 1 m^3^) according to the details given in Fig 1. Each tank has water supplied from the same water sump, treated with UV filter and biofilters. All tanks had their own water supply. Fish were acclimatized for one week before commencement of trial. The duration of this trial was 60 days and all fish were healthy by end of this trial. Survival rate is mentioned in section 2.3. This time period for the trial was selected following [49], which investigated growth of tilapia over a period of 171 days, aiming to achieve a final stocking density of 57.81 kg/m^3^. Furthermore, 60 days is the most recommended study period to observe the effect of any dietary supplement on growth of fish. Duration of trial (60 days) was the only designated humane end point in this study to terminate experiment. Fish husbandry conditions and health were excellently maintained throughout the entire trial period to minimize the mortality as mentioned in section 2.3.

Three stocking density regimes were studied in this trial; low density (LD) (1.50 kg/m^3^), medium density (MD) (3.00 kg/m^3^), high density (HD) (4.50 kg/m^3^). The total number of fish stocked in LD, MD and HD treatment was 600, 1,200 and 1,800, respectively. Each density treatment had three replicates (Fig 1). Fish in all stocking density treatments (LD, MD, HD) were fed with four different levels of dietary supplementation of curcumin (C0, C1, C2, C3). Dose of curcumin in each dietary level is given in section 2.1. Each curcumin supplementation level was studied in further three replicates (Fig 1). These four different levels of curcumin were fed to low density treatment (LDC0, LDC1, LDC2, LDC3), medium density treatment (MDC0, MDC1, MDC2, MDC3), and high-density treatment (HDC0, HDC1, HDC2, HDC3). Fish were fed by hand with daily ration calculated at the rate of 2% of biomass in each replicate. Random weight check of fish was performed for each replicate after every 15 days to adjust the daily ration.

### 2.3 Water quality parameters and survival rate

Water quality was very well maintained in all tanks to ensure the welfare of fish. A total 20% of water was exchanged every day from each tank. Water quality parameters were measured twice a day to ensure its standard quality levels throughout the trial period. Aeration pumps (120V/60Hz, Airmax SilentAir LR25, USA) were used to deliver air via diffuser grids. There was one rectangular diffuser grid in each tank (L ×W: 1 × 0.5 ft). Each diffuser grid was built by using the anti-microbial tubing (outer diameter: 25.4 mm; inner diameter: 12.7 mm; airflow: 2.2 m3/h/meter) to generate microbubbles ensuring the good air saturation in water (>80%). All tanks were back washed on daily basis to drain solid waste settled at the bottom of tank. Water quality parameters including water temperature (25.86 ± 0.30–27.78 ± 0.23°C), dissolved oxygen (4.13 ± 0.31–4.88 ± 0.27 mg/L), and pH (7.52 ± 0.45–8.76 ± 0.01) were monitored twice a day by using portable meters (HI98494, Hanna, USA). Ammonia (0.07±0.03–0.28±0.12 ppm), and nitrite (0.11±0.01–0.21±0.10 mg/L) were monitored twice a week by using commercial kits (HI733, HI93708, Hanna, USA). Fish in every tank were checked for any sign of disease, abnormal behavior and mortality twice a day. Dead fish were removed immediately if found and carefully recorded. Survival rate observed in LD, MD and HD were 100%, 100% and 98.75%, respectively due to well-maintained husbandry conditions over the study period. Mortality of only 1.25% observed in HD treatment, was due to high density. However, it was much lower than the designated limit of 10%, approved by Animal Ethics Committee for Aquaculture trials.

### 2.4. Sample analysis

At end of the trial, five fish were randomly sampled from each replicate (Fig 1) of all density treatments (20 samples per treatments). A total of 180 fish were used for this terminal sampling out of 3600 fish used in the trial following the designated limit of 5% of population for euthanization by Animal Ethics Committee. Remaining 3420 fish were humanely released in nearby lake, administered by Department of Fisheries, Pakistan for stocking purpose (Release Approval #: DOF/27856/2022). Before sampling, fish were fasted for 24 h. On sampling day, they were euthanized using clove oil (0.8 ml/L of water, Sigma-Aldrich, USA). This dose of clove oil is standard to euthanize fish in a very humane way which took less than ten minutes to euthanize sampled fish.

Blood was collected from the caudal vein in two tubes. One tube was coated with ethylenediamine tetra acetic acid (EDTA: (for hematology) while second tube contained clot activator for plasma collection. Blood samples were centrifuged at 5,000 rpm for 15 min and plasma was collected in separate eppendorf tubes and stored at -20°C. The total body weight and total body length were measured before dissection. Fish were dissected, and gills were collected, rinsed in deionized water and preserved in 10% formaldehyde solution for 24 h for histological study. Fish muscle samples were collected and stored at– 20°C for whole body chemical composition. following the guidelines of Association of Official Analytical Chemists (AOAC) [50]. Muscle samples were dehydrated in an oven at 80°C until a consistent dry weight was reached. These dried samples were processed for further chemical analysis. The Kjeldahl apparatus (PCSIR Laboratories, Pakistan) was used to determine the crude protein, while crude lipids were identified using the Folch method [51] using the Soxhlet apparatus (PCSIR Laboratories, Pakistan). The ash content in muscles was determined using muffle furnace (PCSIR Laboratories, Pakistan). The intestinal samples from midgut were weighed, rinsed with deionized water and homogenized in 0.86% sterile normal saline solution (1:9). This mixture was centrifuged at 5000 rpm for 15 min. Supernatant was collected and stored at -20°C. Each sample was analyzed in three replicates for each laboratory analysis. Liver samples were collected and homogenized in liquid nitrogen at -80°C for genes expression analysis. Condition factor (K), specific growth rate (%) (SGR), hepatosomatic index (HSI), fish weight gain, survival rate, feed conversion rate (FCR) was measured by using the given formulae.


$$Condition\;factor\;\left(\%\right)\;=\;\frac{Total\;body\;weight}{Total\;body\;length3}\times 100$$

$$Specific\;growth\;rate\;\left(\%\right)\;=\;\frac{Ln\;final\;weight-Ln\;initial\;weight}{Time\;interval\;in\;days}\times 100$$

$$Hepatosomatic\;index\;=\;\frac{Liver\;weight}{Total\;body\;weight}\times 100$$

$$Feed\;conversion\;ratio\;=\;\frac{Weight\;of\;feed\;consumed}{Weight\;gain\;(wet\;weight)}$$

$$Survival\;rate\;\left(\%\right)\;=\;\frac{Final\;number\;of\;fish}{Initial\;stocking\;density}\times 100$$

### 2.5. Hematological analysis

Hematological parameters such as hemoglobin (Hb) (g/dl), white blood cells (WBC) (10^3^/μL) count such as Neutrophils (%), Eosinophils (%), Lymphocytes (%), Monocytes (%), red blood cell (RBC) (10^6^/μL) count, platelets (10^3^/μL) were determined by using auto-hematology blood analyzer (Sysmex-KX-21, Japan), calibrated for fish.

### 2.6. Biochemical analysis

Triglyceride (TG) (mg/dl) was estimated through a triglyceride colorimetric assay kit (Thermo Fisher Scientific, USA, CAT No. EEA028) following the protocol of the manufacturer. The level of albumin (Alb) (g/dl) was determined through the use of bromocresol green (BCG) dye binding technique, utilizing an albumin kit (LOT. DR379E249; ANMOL-LAB Pvt. Ltd, India). The quantification of alkaline phosphatase (ALP) (U/L) was carried out using commercial kit (Thermo Fisher Scientific, USA, CAT No. EEA002, E.C. 3. I. 3.1.). Aspartate aminotransferase (AST) (U/L) was estimated through commercial ELISA kit (Thermo Fisher Scientific, USA, CAT No. MAK055, E.C. 2.6. 1.1.). Activity of alanine aminotransferase (ALT) (U/L) was measured using commercial ELISA kit (Thermo Fisher Scientific, USA, CAT No. MAK052, E.C. 2.6. 1.2.). The concentration of glucose (GLU) (mg/dl) was measured by using laboratory blood glucose analyzer (Human, Germany).

### 2.7. Cortisol assay

The concentration of cortisol (ng/ml) in blood plasma was measured using ELISA (Calbiotech, USA, CAT No. CO368S, CID 5754) having a sensitivity of 1.16 ng/ml. The intra-assay and inter-assay precision were 3.8% and 8.65%, respectively. The detection limit was 0–800 ng/ml. The absorbance value was read on spectrophotometer at 450 nm.

### 2.8. Antioxidants assay

Plasma catalase (CAT) (U/ml) activity was determined using a commercial ELISA colorimetric activity kit (Thermo Fisher Scientific, USA, CAT No. EIACATC, *EC 1*.*11*.*1*.*6*) having an analytical sensitivity of 0.052 U/ml. The absorbance was read at 560 nm at 37°C. The activity of superoxide dismutase (SOD) (ng/ml) were measured by using ELISA kit (PARS BIOCHME, China, CAT No. PRS-02005hu, EC 1.15.1.1) with an assay range of 0.3 ng/ml– 10 ng/ml. Malondialdehyde (MDA) (nmol/ml) ELISA kit (PARS BIOCHME, China, CAT No. PRS-00991hu, CAS 542-78-9) with an assay range of 0.3 ng/ml– 7 nmol/ml. Activity of glutathione peroxidase (GPx) (IU/ml) were measured by using ELISA kit (PARS BIOCHME, China, CAT No. PRS-00680hu, EC 1.11. 1.9) with an assay range of 3 IU/ml– 200 IU/ml. The absorbance value of SOD, MDA, and GPx was read at 450 nm and 37°C.

### 2.9. Digestive enzymes assay

For digestive enzyme analyses, the supernatant of processed whole intestine samples was utilized. Activity of lipase (U/L) was assayed with a commercial ELISA kit (Sigma Aldrich, USA, CAT No. MAK046, EC 3.1.1.3) with a detection limit of 5 U/L to 250 U/L at 37°C and 570 nm of wavelength. Amylase (U/L) activity was measured using a commercial ELISA kit (Sigma Aldrich, USA, CAT No. *MAK009A*, EC 3.2. 1.1.) with a detection limit of 0.2439 U/L—2200 U/L at 37°C and 405 nm of wavelength. The activity of protease was determined following instructions [52]. Casein 1% w/v was used as substrate in 0.2 M phosphate buffer at pH 7.0. One unit of protease indicates the amount of enzyme that releases 1 μg/ml/min of tyrosine determined at 660 nm of wavelength.

### 2.10. Histological study

Preserved gill samples were dehydrated by passing through different grades of alcohol (70%, 90% and 100%) and xylene. For the infiltration of wax, gills were processed in paraffin wax. Microtome were used for sectioning and wax blocks were trimmed at 10 μ and then transverse sections of 4 μ thickness were cut. For dewaxing, xylene and alcohol were used and stained with haematoxyline and eosin. Stained section of gills was mounted with DPX (mixture of distyrene, plasticizer and xylene) (Merck, Germany). Microphotographs were taken at digital camera fitted optical microscope (Trinocular E-200, Nikon Japan Eil-12).

### 2.11. Gene expression analysis

Liver samples (50 mg/sample) were used to extract total RNA by using trizol (Catalog No. 15596026, Thermo, USA) method at 37°C. The quality and quantity of each sample was verified on Nanodrop 2000 spectrophotometer (Thermo, Waltham, MA, USA). The first strand cDNA was synthesized using super script III first strand cDNA synthesis kit (Cat No. 18080051, Life technologies). The 5.0 μg of total RNA was used for cDNA synthesis. cDNA synthesis was performed in the first step with poly-A tail primedoligodT in a total volume of 20 μl. The first reaction mixture was prepared having RNA 5 μg, 50 μMoligo (dT) 20 of 1 μL, 10 mMdNTP mix of 1 μL and then water was added upto 10 μL. The mixture was incubated at 65°C for 5 min. cDNA synthesis Mix-2 was prepared by adding 10 X RT buffer (2 μL), 25 mM MgCl_2_ (4 μL), 0.1 M DTT (2 μL), RNaseOUT™ (40 U/μL) (1 μL), SuperScript® III RT (200 U/μL) (1 μL), a total of 10 μL.

The prepared 10 μL of cDNA Synthesis mix was added to each RNA/primer mixture, mixed gently and collected by brief centrifugation. The tube was incubated for 50 min at 50°C. The reaction was terminated at 85°C for 5 min. The cDNA was store at -20°C. The PCR reaction was performed in a separate tube with gene specific primers (forward and reverse) using 2 μl cDNA templates. The following set of primer was used for real-time PCR which were designed by using software Primer Quest from integrated DNA technology (Table 2). Each set of primer 1 μl (10 μM) along with 12.5 μl SYBR green PCR master mix (Maxima SYBR Green/ROX qPCR Master Mix (2X)) were used. First denaturation step was carried out as 95°C for 2 min, followed by 95°C denaturation for 15 sec, annealing step was carried out at 55°C for 1 min and extension step was carried out at 72°C for 1 min. β-actin was used as the housekeeping gene for reference. The 2-fold induction was determined by ΔΔCT method (relative quantification).

**Table 2:** Primer sequence of genes.

#	Gene	Sequence 5 to 3
01	Somatostatin-1 (SST-1) F	TGCTGGGCTCCAAACAG
02	Somatostatin-1 (SST-1) R	AGGGAAGTTCTCCTCTTCCA
03	Interleukin 1-β (IL-1β) F	TGGAGGAGGTGACGGATAAA
04	Interleukin 1-β (IL-1β) R	CAGTGTCGCGTTTGTAGAAGA
05	Proopiomelanocortin (POMC-α) F	CTCCTACTCAATGGAGCACTTC
06	Proopiomelanocortin (POMC-α) R	AAGCTCTCGTCTCCTCATCT
07	β-Actin-F	GAGGTATCCTGACCCTGAAGTA
08	Β-Actin-R	ACTCTCAGCTCGTTGTAGGA

### 2.12. Statistical analysis

For all the statistical analyses, SPSS v.29 software was used. Data were presented as Mean± SE for all the parameters. Levene test were performed to check the homogeneity of variance of data. The effect of density and curcumin supplementation dose on different parameters was determined by Two-Way ANOVA. To reject the null hypothesis, 0.05 probability level was used. Superscripts represented as upper case letters show the comparison (P < 0.05) between three density treatments (LDC, MDC, HDC). Superscripts represented as lower-case letters (red color) show the comparison (P < 0.05) between four different levels of curcumin supplementation within one density treatment.

## 3. Results

### 3.1 Water quality parameters

The range of water quality parameters including water temperature (25.86 ± 0.30–27.78 ± 0.23°C), dissolved oxygen (4.13 ± 0.31–4.88 ± 0.27 mg/L), and pH (7.52 ± 0.45–8.76 ± 0.01), ammonia (0.07±0.03–0.28±0.12 ppm), and nitrite (0.11±0.01–0.21±0.10 mg/L) (Table 3).

**Table 3:** Mean value of physiochemical parameters (water temperature (°C), dissolved oxygen (mg/L), pH, ammonia (ppm)and nitrites (mg/L) measured in all groups.

Groups	Water temperature (°C)	Dissolved oxygen (mg/L)	pH	Ammonia (ppm)	Nitrites (mg/L)
**LC0**	27.20 ± 0.32	4.56 ± 0.36	7.52 ± 0.45	0.09±0.02	0.11±0.01
**LC1**	26.30 ± 0.32	4.54 ± 0.42	8.30 ± 0.06	0.07±0.03	0.12±0.03
**LC2**	26.31 ± 0.32	4.51 ± 0.45	8.60 ± 0.02	0.08±0.04	0.15±0.04
**LC3**	27.40 ± 0.28	4.64 ± 0.31	8.40 ± 0.01	0.11±0.05	0.18±0.018
**MC0**	27.44 ± 0.30	4.88 ± 0.27	8.75 ± 0.04	0.10±0.04	0.15±0.08
**MC1**	26.88 ± 0.25	4.43 ± 0.31	7.80 ± 0.11	0.12±0.08	0.16±0.07
**MC2**	27.63 ± 0.22	4.65 ± 0.36	8.22 ± 0.45	0.13±0.09	0.17±0.03
**MC3**	26.53 ± 0.28	4.59 ± 0.42	8.49 ± 0.04	0.09±0.08	0.12±0.03
**HC0**	26.61 ± 0.29	4.73 ± 0.45	8.70 ± 0.03	0.28±0.12	0.14±0.06
**HC1**	27.78 ± 0.23	4.74 ± 0.31	8.60 ± 0.02	0.27±0.16	0.16±0.06
**HC2**	26.98 ± 0.22	4.49 ± 0.27	8.76 ± 0.01	0.23±0.13	0.19±0.09
**HC3**	25.86 ± 0.30	4.13 ± 0.31	8.43 ± 0.11	0.22±0.13	0.21±0.10

### 3.2. Growth

In three different densities treatment (LDC, MDC, HDC), total body length (df_2_, F = 14.03, P = 0.0000), total body weight (df_2_, F = 6.88, P = 0.0024), condition factor (df_2_, F = 16.31, P = 0.0000), specific growth rate (df_2_, F = 7.39, P = 0.0016), hepatosomatic index (df_2_, F = 15.72, P = 0.0000) were significantly different (Table 4). A significant variation in total body length (df_3_, F = 4.25, P = 0.01), condition factor (df_3,_ F = 3.54, P = 0.02), specific growth rate (df_3,_ F = 2.79, P = 0.05) and hepatosomatic index (df_3,_ F = 0.79, P = 0.50), except total body weight (df_3_, F = 2.62, P = 0.06) were observed between different levels of curcumin supplementation in all three-density treatment (levels of supplementation: 04 in each treatment). Other than this, effect of density*curcumin calculated by two-way ANOVA also showed a significant effect on total body length (df_6_, F = 2.67, P = 0.03), total body weight (df_6_, F = 2.58, P = 0.03), condition factor (df_6_, F = 2.47, P = 0.04), specific growth rate (df_6_, F = 2.85, P = 0.02) except hepatosomatic index on which the effect was insignificant (df_6_, F = 1.69, P = 0.14). The survival rate of fish both in LSD and MSD was 100% but in HSD the its rate was 98.22% - 99.56%.

**Table 4:** Analysis of total body length (cm), total body weight (g), condition factor, hepatosomatic index, specific growth rate and FCR (Mean ± SE) in three density treatment (LDC, MDC, HDC) having four curcumin supplementation levels (C0 = 0 mg/kg, C1 = 50 mg/kg, C2 = 100 mg/kg, C3 = 150 mg/kg). Superscripts represented as upper case letters show the comparison (P < 0.05) between three density treatments (LDC, MDC, HDC). Superscripts represented as lower-case letters show the comparison (P < 0.05) between four different levels of curcumin supplementation within one density treatment.

Treatments	TBL (cm)	TBW (g)	SGR (%)	K (%)	HSI (%)	FCR	Survival rate (%)
**LDC0**	7.18±0.24 ^A,a^	74.00±1.10 ^AB,a^	423.39±2.24 ^AB,a^	20.12±0.29 ^B,b^	0.96±0.03 ^A,a^	0.82±0.39 ^B,d^	100.00±0.00 ^B,c^
**LDC1**	9.18±0.29 ^A,b^	95.75±0.99 ^AB,a^	447.50±1.44 ^AB,b^	14.40±0.55 ^B,a^	0.97±0.04 ^A,a^	0.55±0.44 ^B,a^	100.00±0.00 ^B,b^
**LDC2**	8.75±0.34 ^A,ab^	93.00±1.21 ^AB,a^	446.28±2.62 ^AB,ab^	15.04±0.66 ^B,ab^	0.98±0.05 ^A,a^	0.57±0.43 ^B,b^	100.00±0.00 ^B,a^
**LDC3**	8.98±0.44 ^A,ab^	92.25±1.11 ^AB,a^	445.90±1.66 ^AB,ab^	13.08±0.84 ^B,ab^	1.03±0.03 ^A,a^	0.58±0.43 ^B,c^	100.00±0.00 ^B,d^
**MDC0**	9.95±0.34 ^B,a^	98.75±1.23 ^B,a^	453.42±1.43 ^B,a^	10.05±0.67 ^A,b^	1.07±0.02 ^B,a^	0.52±0.54 ^A,d^	100.00±0.00 ^B,c^
**MDC1**	10.05±0.28 ^B,b^	95.50±1.32 ^B,a^	450.00±1.54 ^B,b^	9.42±0.54 ^A,a^	1.11±0.04 ^B,a^	0.55±0.64 ^A,a^	100.00±0.00 ^B,b^
**MDC2**	9.63±0.29 ^B,ab^	94.75±1.77 ^B,a^	449.21±1.64 ^B,ab^	10.71±0.34 ^A,ab^	1.12±0.03 ^B,a^	0.56±0.43 ^A,b^	100.00±0.00 ^B,a^
**MDC3**	8.63±0.24 ^B,ab^	84.00±1.88 ^B,a^	437.16±1.78 ^B,ab^	13.18±0.56 ^A,ab^	1.03±0.03 ^B,a^	0.67±0.29 ^A,c^	100.00±0.00 ^B,d^
**HDC0**	7.60±0.33 ^A,a^	77.00±1.24 ^A,a^	428.42±1.87 ^A,a^	17.73±0.59 ^B,b^	0.97±0.05 ^A,a^	0.77±0.39 ^C,d^	98.89±0.06 ^A,c^
**HDC1**	8.88±0.29 ^A,b^	86.50±1.66 ^A,a^	440.02±1.64 ^A,b^	12.68±0.67 ^B,a^	1.02±0.04 ^A,a^	0.64±0.44 ^C,a^	98.44±0.08 ^A,b^
**HDC2**	7.95±0.34 ^A,ab^	82.00±1.69 ^A,a^	434.28±1.44 ^A,ab^	17.05±0.76 ^B,ab^	0.99±0.03 ^A,a^	0.69±0.43 ^C,b^	98.22±0.09 ^A,a^
**HDC3**	7.98±0.30 ^A,ab^	81.00±1.59 ^A,a^	433.28±1.77 ^A,ab^	16.64±0.49 ^B,ab^	0.99±0.05 ^A,a^	0.71±0.32 ^C,c^	99.56±0.05 ^A,d^

### 3.3. Chemical composition of muscles

The moisture content (df_2_, F = 408.67), crude protein (df_2_, F = 756.29), crude ash (df_2_, F = 1641.52) and crude fat (df_2_, F = 2251.88) was significantly different (P = 0.0000) between three density treatment (Table 5). A significant difference (P = 0.0000) in the content of moisture (df_3_, F = 1746.03), crude protein (df_3_, F = 3392.86), crude ash (df_3_, F = 3549.21) and crude fat (df_3_, F = 3013.29) was observed between different levels of curcumin supplementation in all three-density treatment (levels of supplementation: 04 in each treatment). Effect of density*curcumin calculated by Two-way ANOVA also showed significant effect (P = 0.0000) on moisture (df_6_, F = 1924.60), crude protein (df_6_, F = 2440.48), crude ash (df_6_, F = 1596.83) and crude fat (df_6_, F = 3328.77).

**Table 5:** Analysis (Means ± SE) of chemical composition (%) of muscles in three density treatment (LDC, MDC, HDC) having four curcumin supplementation levels (C0 = 0 mg/kg, C1 = 50 mg/kg, C2 = 100 mg/kg, C3 = 150 mg/kg). Superscripts represented as upper case letters show the comparison (P < 0.05) between three density treatments (LDC, MDC, HDC). Superscripts represented as lower-case letters show the comparison (P < 0.05) between four different levels of curcumin supplementation within one density treatment.

Treatments	Moisture (%)	Crude protein (%)	Crude fat (%)	Crude ash (%)
**LDC0**	74.00±0.99 ^B,c^	18.60±0.24 ^B,a^	1.80±0.11 ^B,b^	5.60±0.07 ^B,c^
**LDC1**	74.10±0.98 ^B,b^	18.50±0.33 ^B,c^	1.90±0.12 ^B,b^	5.70±0.06 ^B,b^
**LDC2**	73.90±0.88 ^B,a^	18.70±0.43 ^B,d^	1.87±0.13 ^B,c^	5.54±0.07 ^B,a^
**LDC3**	74.30±0.79 ^B,b^	18.90±0.54 ^B,b^	1.20±0.15 ^B,a^	5.60±0.07 ^B,d^
**MDC0**	74.10±0.87 ^C,c^	18.40±0.45 ^A,a^	1.70±0.14 ^C,b^	5.80±0.06 ^A,c^
**MDC1**	74.20±0.98 ^C,b^	18.70±±0.65 ^A,c^	1.60±0.13 ^C,b^	5.50±0.06 ^A,b^
**MDC2**	74.10±0.89 ^C,a^	18.80±0.67 ^A,d^	1.90±0.11 ^C,c^	5.20±0.05 ^A,a^
**MDC3**	74.00±0.88 ^C,b^	18.50±0.87 ^A,b^	1.80±0.14 ^C,a^	5.70±0.06 ^A,d^
**HDC0**	74.30±0.89 ^A,c^	18.50±0.24 ^C,a^	1.50±0.15 ^A,b^	5.70±0.04 ^C,c^
**HDC1**	74.00±0.99 ^A,b^	18.90±0.34 ^C,c^	1.50±0.13 ^A,b^	5.60±0.03 ^C,b^
**HDC2**	73.80±1.11 ^A,a^	18.90±0.43 ^C,d^	1.70±0.12 ^A,c^	5.60±0.05 ^C,a^
**HDC3**	74.00±1.21 ^A,b^	18.50±0.43 ^C,b^	1.60±0.17 ^A,a^	5.90±0.04 ^C,d^

### 3.4. Digestive enzymes activity

**Fig 2:**
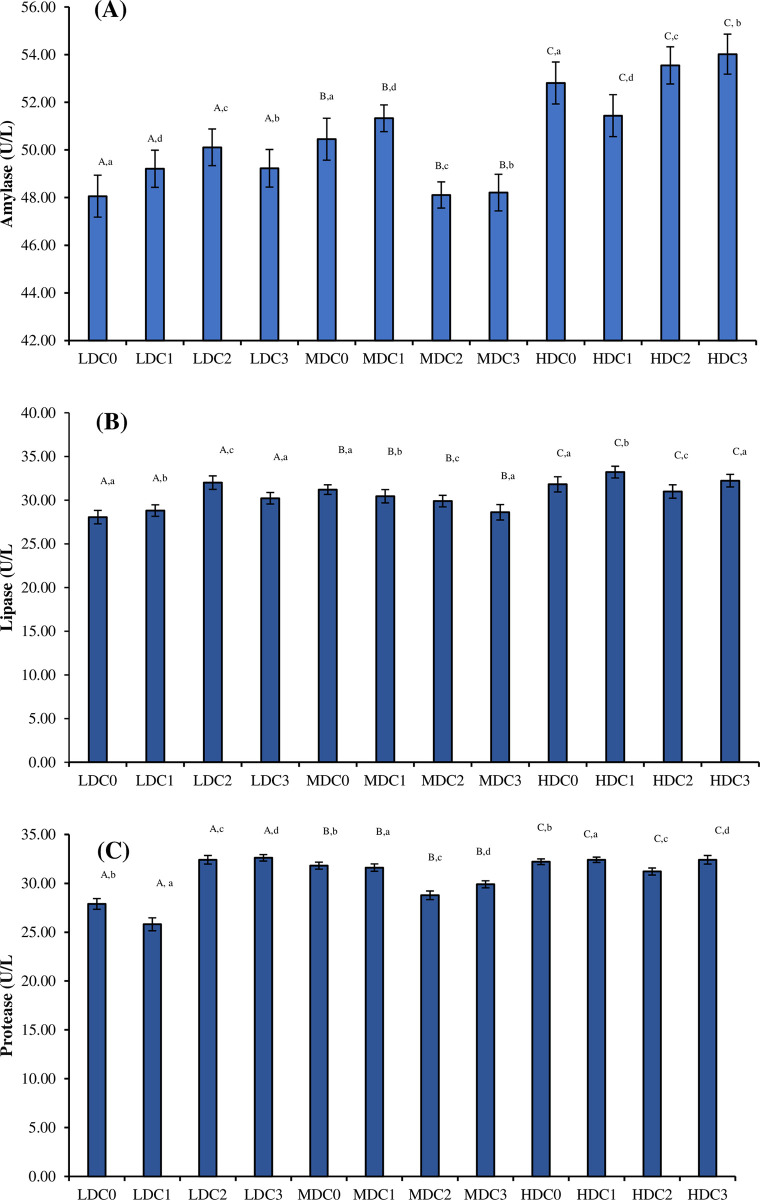
Levels of (A) amylase, (B) lipase and (C) protease (Mean ± SE) determined in three density treatment (LDC, MDC, HDC) having four curcumin supplementation levels (C0 = 0 mg/kg, C1 = 50 mg/kg, C2 = 100 mg/kg, C3 = 150 mg/kg). Superscripts represented as upper case letters show the comparison (P < 0.05) between three density treatments (LDC, MDC, HDC). Superscripts represented as lower-case letters show the comparison (P < 0.05) between four different levels of curcumin supplementation within one density treatment.

The activity of amylase (df_2_, F = 1256956.88), protease (df_2_, F = 417165.81) and lipase (df_2_, F = 447251.52) were significantly different (P = 0.0000) between three density treatment (LDC, MDC, HDC) (Fig 2). Different levels of curcumin supplementation in density treatment (four in each treatment) showed significant variations (P = 0.0000) in the activity of amylase (df_3_, F = 2119.64), lipase (df_3_, F = 21273.81) and protease (df_3_, F = 104146.83). In addition to this, effect of density*curcumin on profile of amylase (df_6_, F = 162860.12), lipase (df_6_, F = 170659.52) and protease (df_6_, F = 441651.60) calculated by Two-way ANOVA was also significant (P = 0.0000).

### 3.5. Profile of cortisol

**Fig 3:**
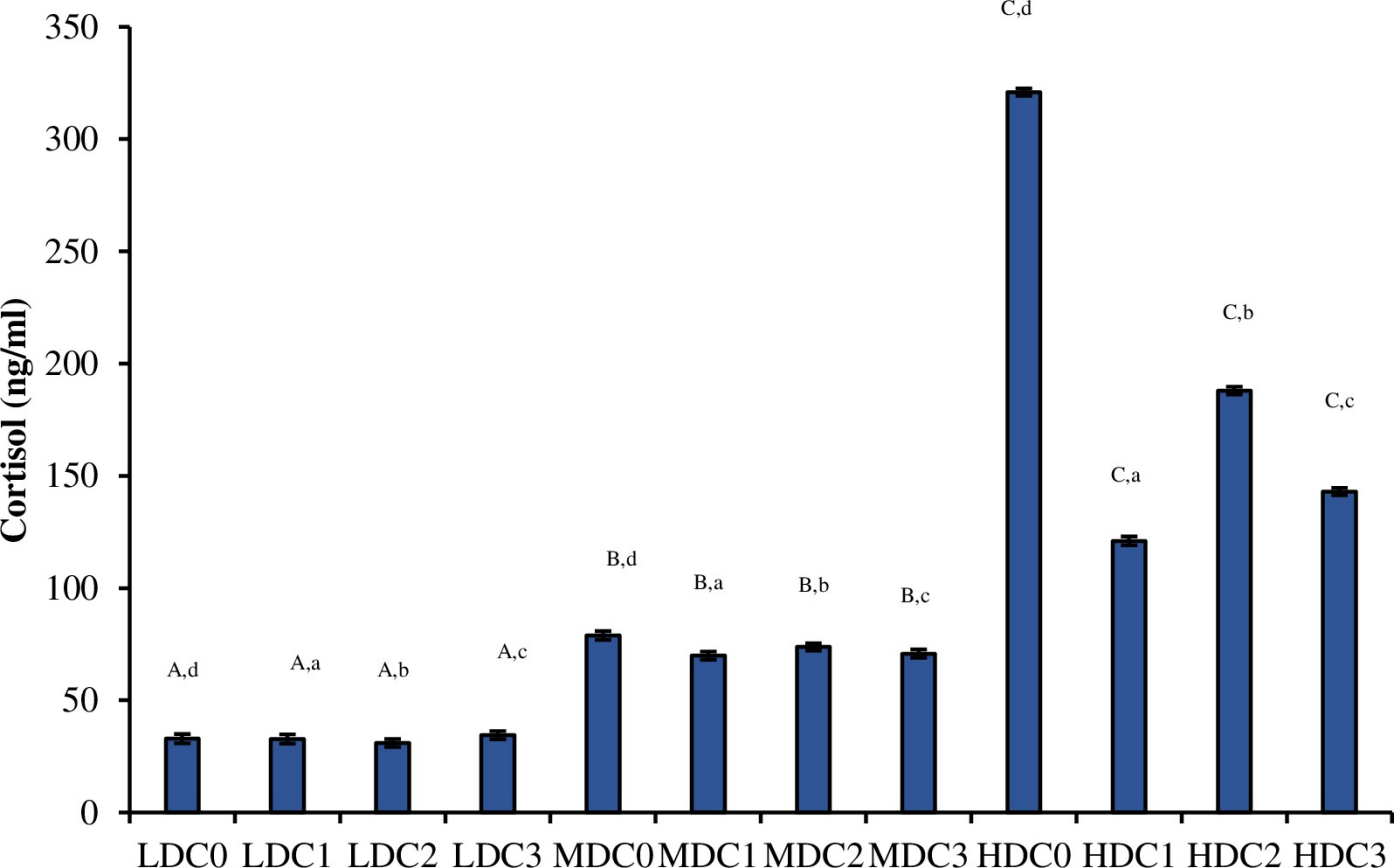
Level of cortisol (Mean ± SE) determined in three density treatment (LDC, MDC, HDC) having four curcumin supplementation levels (C0 = 0 mg/kg, C1 = 50 mg/kg, C2 = 100 mg/kg, C3 = 150 mg/kg). Superscripts represented as upper case letters show the comparison (P < 0.05) between three density treatments (LDC, MDC, HDC). Superscripts represented as lower-case letters show the comparison (P < 0.05) between four different levels of curcumin supplementation within one density treatment.

The levels of cortisol differed significantly (df_2_, F = 560177080.92, P = 0.0000) between density treatment (LDC, MDC, HDC) (Fig 3). Effect of different levels of curcumin supplementation in all three-density treatment (four in each treatment) was also significant (df_3_, F = 39657792.65, P = 0.0000). Effect of density*curcumin on the level of cortisol calculated by Two-way ANOVA was also significant (df_6_, F = 29618622.42, P = 0.0000).

### 3.6. Blood biochemistry and hematology

A significant effect (P = 0.0000) was observed on the content of Hb (df_2_, F = 14772.95), platelets (df_2_, F = 102144144.38), WBC (df_2_, F = 78.05), RBC (df_2_, F = 3262.60), monocytes (df_2_, F = 2984.85), eosinophils (df_2_, F = 380.09), neutrophils (df_2_, F = 792620.57) and lymphocytes (df_2_, F = 872715.80) in three density treatment (Table 6). Content of triglycerides (df_2_, F = 5456572.95), ALT (df_2_, F = 6292715.80), AST (df_2_, F = 60185715.80), ALP (df_2_, F = 1251790858.66), albumin (df_2_, F = 47058.66), glucose (df_2_, F = 14722334.85), urea (df_2_, F = 14010430.09) showed a significant effect (P = 0.0000) in three density treatment except in creatinine (df_2_, F = 1.52) which was insignificantly different (P = 0.23) (Table 7).

**Table 6:** Analysis (Means ± SE) of blood hematological (Mean ± SE) in plasma samples in three density treatment (LDC, MDC, HDC) having four curcumin supplementation levels (C0 = 0 mg/kg, C1 = 50 mg/kg, C2 = 100 mg/kg, C3 = 150 mg/kg). Superscripts represented as upper case letters show the comparison (P < 0.05) between three density treatments (LDC, MDC, HDC). Superscripts represented as lower-case letters show the comparison (P < 0.05) between four different levels of curcumin supplementation within one density treatment.

Treatments	Hemoglobin (g/dl)	Total RBC (× 106/μL)	Platelets (× 103/μL)	WBC (× 103/μL)	Neutrophils (%)	Lymphocytes (%)	Monocytes (%)	Eosinophils (%)
**LDC0**	7.10±0.13 ^A,d^	1.07±0.06 ^B,d^	144.00±0.55 ^A,d^	34.35±0.16 ^B,b^	86.00±0.39 ^B,a^	14.00±0.54 ^C,b^	2.00±0.01 ^A,a^	2.30±0.03 ^C,d^
**LDC1**	6.60±0.14 ^A,a^	0.89±0.07 ^B,b^	88.00±0.45 ^A,a^	30.00±0.18 ^B,a^	81.00±0.42 ^B,d^	19.00±0.53 ^C,a^	2.00±0.02 ^A,b^	2.20±0.03 ^C,c^
**LDC2**	6.20±0.12 ^A,b^	0.90±0.05 ^B,c^	98.00±0.43 ^A,c^	31.00±0.17 ^B,a^	81.00±0.40 ^B,b^	19.00±0.45 ^C,c^	2.00±0.02 ^A,b^	2.30±0.03 ^C,a^
**LDC3**	6.00±0.11 ^A,c^	0.97±0.06 ^B,a^	81.00±0.45 ^A,b^	32.00±0.18 ^B,a^	78.00±0.55 ^B,c^	22.00±0.53 ^C,c^	2.00±0.03 ^A,b^	2.30±0.02 ^C,b^
**MDC0**	7.00±0.16 ^B,d^	1.08±0.09 ^C,d^	123.00±0.55 ^B,d^	32.33±0.19 ^A,b^	71.00±0.51 ^A,a^	17.00±0.55 ^B,b^	2.00±0.04 ^A,a^	2.40±0.04 ^A,d^
**MDC1**	6.20±0.17 ^B,a^	1.00±0.07 ^C,b^	111.00±0.59 ^B,a^	27.00±0.21 ^A,a^	88.00±0.54 ^A,d^	12.00±0.65 ^B,a^	2.00±0.05 ^A,b^	2.30±0.06 ^A,c^
**MDC2**	6.70±0.16 ^B,b^	1.10±0.09 ^C,c^	113.00±0.69 ^B,c^	24.00±0.23 ^A,a^	81.00±0.65 ^A,b^	19.00±0.76 ^B,c^	2.00±0.04 ^A,b^	2.10±0.06 ^A,a^
**MDC3**	6.80±0.19 ^B,c^	0.98±0.08 ^C,a^	116.00±0.77 ^B,b^	23.00±0.31 ^A,a^	85.00±0.76 ^A,c^	15.00±0.85 ^B,c^	2.00±0.03 ^A,b^	2.00±0.08 ^A,b^
**HDC0**	6.60±0.22 ^C,d^	1.09±0.09 ^A,d^	165.00±0.70 ^C,d^	30.37±0.33 ^B,b^	84.00±0.67 ^C,a^	16.00±0.87 ^A,b^	2.10±0.09 ^C,a^	2.40±0.07 ^B,d^
**HDC1**	6.90±0.23 ^C,a^	0.79±0.07 ^A,b^	121.00±0.71 ^C,a^	32.46±0.36 ^B,a^	86.00±0.68 ^C,d^	14.00±0.88 ^A,a^	2.20±0.08 ^C,b^	2.20±0.06 ^B,c^
**HDC2**	7.00±0.23 ^C,b^	0.76±0.06 ^A,c^	131.00±0.73 ^C,c^	31.48±0.34 ^B,a^	83.00±0.69 ^C,b^	15.00±0.69 ^A,c^	2.20±0.07 ^C,b^	2.10±0.05 ^B,a^
**HDC3**	7.20±0.27 ^C,c^	0.67±0.07 ^A,a^	143.00±0.79 ^C,b^	35.07±0.35 ^B,a^	84.00±0.66 ^C,c^	16.00±0.78 ^A,c^	2.20±0.05 ^C,b^	2.30±0.05 ^B,b^

**Table 7:** Analysis on blood biochemistry (Mean ± SE) in plasma samples in plasma samples in three density treatment (LDC, MDC, HDC) having four curcumin supplementation levels (C0 = 0 mg/kg, C1 = 50 mg/kg, C2 = 100 mg/kg, C3 = 150 mg/kg). Superscripts represented as upper case letters show the comparison (P < 0.05) between three density treatments (LDC, MDC, HDC). Superscripts represented as lower-case letters show the comparison (P < 0.05) between four different levels of curcumin supplementation within one density treatment.

Treatments	Triglycerides (mg/dl)	Glucose (mg/dl)	ALT (U/L)	AST (U/L)	Alkaline Phosphate (U/L)	Albumin (g/dl)	Urea (mg/dl)	Creatinine (mg/dl)
**LDC0**	190.00±0.77 ^A,d^	72.00±0.87 ^B,d^	19.00±0.88 ^B,c^	12.00±0.66 ^A,d^	75.00±0.35 ^A,d^	0.90±0.01 ^C,a^	16.00±0.12 ^B,d^	0.50±0.03 ^A,c^
**LDC1**	188.00±0.87 ^A,a^	70.00±0.89 ^B,b^	22.00±0.76 ^B,a^	14.00±0.62 ^A,a^	80.00±0.31 ^A,a^	2.30±0.03 ^C,c^	15.00±0.14 ^B,b^	0.80±0.04 ^A,d^
**LDC2**	192.00±0.88 ^A,c^	71.00±0.78 ^B,c^	35.00±0.67 ^B,d^	19.00±0.65 ^A,b^	84.00±0.36 ^A,b^	2.00±0.02 ^C,d^	12.00±0.13 ^B,a^	0.40±0.02 ^A,b^
**LDC3**	188.00±0.89 ^A,b^	73.00±0.79 ^B,a^	28.00±0.81 ^B,b^	22.00±0.76 ^A,c^	77.00±0.44 ^A,c^	3.20±0.07 ^C,b^	20.00±0.12 ^B,c^	0.60±0.05 ^A,a^
**MDC0**	200.00±0.99 ^B,d^	80.00±0.87 ^A,d^	26.00±0.80 ^A,c^	49.00±0.66 ^B,d^	99.00±0.48 ^B,d^	1.20±0.08 ^A,a^	15.00±0.18 ^A,d^	0.60±0.06 ^A,c^
**MDC1**	180.00±0.88 ^B,a^	60.00±0.85 ^A,b^	28.00±0.78 ^A,a^	33.00±0.76 ^B,a^	88.00±0.55 ^B,a^	1.40±0.09 ^A,c^	18.00±0.16 ^A,b^	0.80±0.05 ^A,d^
**MDC2**	199.00±0.98 ^B,c^	66.00±0.76 ^A,c^	20.00±0.85 ^A,d^	40.00±0.70 ^B,b^	94.00±0.76 ^B,b^	1.80±0.07 ^A,d^	13.00±0.17 ^A,a^	0.60±0.08 ^A,b^
**MDC3**	189.00±0.88 ^B,b^	63.00±0.70 ^A,a^	17.00±0.81 ^A,b^	39.00±0.67 ^B,c^	101.00±0.67 ^B,c^	1.00±0.09 ^A,b^	16.00±0.16 ^A,c^	0.30±0.09 ^A,a^
**HDC0**	226.00±0.77 ^C,d^	112.00±0.74 ^C,d^	39.00±0.77 ^C,c^	87.00±0.76 ^C,d^	288.00±0.76 ^C,d^	1.80±0.04 ^B,a^	32.00±0.12 ^C,d^	0.70±0.08 ^A,c^
**HDC1**	190.00±0.80 ^C,a^	77.00±0.73 ^C,b^	22.00±0.74 ^C,a^	31.00±0.73 ^C,a^	170.00±0.78 ^C,a^	2.20±0.05 ^B,c^	25.00±0.15 ^C,b^	0.40±0.09 ^A,d^
**HDC2**	187.00±0.86 ^C,c^	74.00±0.72 ^C,c^	32.00±0.73 ^C,d^	28.00±0.76 ^C,b^	169.00±0.76 ^C,b^	2.60±0.06 ^B,d^	28.00±0.17 ^C,a^	0.70±0.05 ^A,b^
**HDC3**	189.00±0.81 ^C,b^	62.00±0.87 ^C,a^	35.00±0.74 ^C,b^	27.00±0.73 ^C,c^	177.00±0.70 ^C,c^	1.40±0.08 ^B,b^	26.00±0.16 ^C,c^	0.50±0.04 ^A,a^

Effect of different levels of curcumin supplementation in all three-density treatment was also studied on hematology and biochemical parameters (four levels in each treatment). A significant effect of this supplementation was observed (P = 0.0000) on Hb (df_3_, F = 4503.96), WBC (df_3_, F = 12.81), RBC (df_3_, F = 1900.79), platelets (df_3_, F = 59515873.01), neutrophils (df_3_, F = 825396.82), lymphocytes (df_3_, F = 404761.90), monocytes (df_3_, F = 59.52), eosinophils (df_3_, F = 1646.82), triglycerides (df_3_, F = 15706349.20), glucose (df_3_, F = 21190476.19), ALT (df_3_, F = 1005952.38), AST (df_3_, F = 24609126.98), ALP (df_3_, F = 80325396.82), albumin (df_3_, F = 28015.87), urea (df_3_, F = 492063.49) and creatinine (df_3_, F = 1488.09).

A significant effect (P = 0.0000) of density*curcumin calculated by Two-way ANOVA were observed on the content of Hb (df_6_, F = 12539.68), WBC (df_6_, F = 17.64), RBC (df_6_, F = 606.15), platelets (df_6_, F = 12783730.15), neutrophils (df_6_, F = 1998015.87), lymphocytes (df_6_, F = 541666.66), monocytes (df_6_, F = 59.52), eosinophils (df_6_, F = 1051.58), triglycerides (df_6_, F = 7944444.44), glucose (df_6_, F = 8767857.14), ALT (df_6_, F = 4101190.47), AST (df_6_, F = 20484126.98), ALP (df_6_, F = 82182539.68), albumin (df_6_, F = 31825.39), urea (df_6_, F = 640873.01) and creatinine (df_6_, F = 2619.04).

### 3.7. Antioxidant assay

A significant difference (P = 0.0000) was observed in the levels of CAT (df_2_, F = 76.19), SOD (df_2_, F = 21.15), GPx (df_2_, F = 654.52) and MDA (df_2_, F = 719.06) between three different density treatment (Table 8). A significant variation in the levels of CAT (df_3_, F = 10.03, P = 0.0000), SOD (df_3_, F = 6.39, P = 0.0010), GPx (df_3_, F = 68.03, P = 0.0000) and MDA (df_3_, F = 13.42, P = 0.0000) were observed between different levels of curcumin supplementation in three density treatment (four in each treatment). Effect of density*curcumin calculated by Two-way ANOVA on the levels of CAT (df_6_, F = 11.02, P = 0.0000), SOD (df_6_, F = 2.84, P = 0.01), GPx (df_6_, F = 67.46, P = 0.0000) and MDA (df_6_, F = 13.42, P = 0.0000) was significant.

**Table 8:** Effect on antioxidant activity Mean±SE) including catalase, superoxide dismutase and glutathione peroxidase and malondialdehyde in plasma samples in three density treatment (LDC, MDC, HDC) having four curcumin supplementation levels (C0 = 0 mg/kg, C1 = 50 mg/kg, C2 = 100 mg/kg, C3 = 150 mg/kg). Superscripts represented as upper case letters show the comparison (P < 0.05) between three density treatments (LDC, MDC, HDC). Superscripts represented as lower-case letters show the comparison (P < 0.05) between four different levels of curcumin supplementation within one density treatment.

Treatments	Catalase	SOD	GPX	MDA
	(U/ml)	(ng/ml)	(IU/ml)	(nmol/ml)
**LDC0**	13.9±0.10 ^A,b^	2.05±0.08 ^A, b^	24.80±0.21 ^B,b^	0.26±0.02 ^A,a^
**LDC1**	12.2±0.11 ^A,ab^	0.82±0.07 ^A,ab^	23.60±0.22 ^B,b^	0.27±0.06 ^A,ab^
**LDC2**	14.33±0.12 ^A,a^	0.80±0.06 ^A,a^	21.50±0.26 ^B,a^	0.28±0.07 ^A,b^
**LDC3**	14.23±0.16 ^A,a^	1.23±0.08 ^A,a^	24.80±0.33 ^B,a^	0.27±0.06 ^A,c^
**MDC0**	14.9±0.17 ^A,b^	2.91±0.09 ^A,b^	25.01±0.32 ^A,b^	0.33±0.05 ^A,a^
**MDC1**	13.00±0.15 ^A,ab^	1.32±0.06 ^A,ab^	22.66±0.31 ^A,b^	0.34±0.06 ^A,ab^
**MDC2**	13.88±0.13 ^A,a^	1.54±0.08 ^A,a^	23.10±0.28 ^A,a^	0.37±0.05 ^A,b^
**MDC3**	14.00±0.15 ^A,a^	2.00±0.08 ^A,a^	25.70±0.26 ^A,a^	0.32±0.06 ^A,c^
**HDC0**	17.3±0.17 ^B,b^	3.22±0.07 ^B,b^	27.88±0.28 ^C,b^	0.48±0.04 ^B,a^
**HDC1**	13.44±0.16 ^B,ab^	2.30±0.08 ^B,ab^	24.40±0.25 ^C,b^	0.29±0.07 ^B,ab^
**HDC2**	15.66±0.15 ^B,a^	2.55±0.09 ^B,a^	25.60±0.27 ^C,a^	0.44±0.05 ^B,b^
**HDC3**	16.33±0.14 ^B,a^	2.88±0.08 ^B,a^	25.50±0.33 ^C,a^	0.28±0.06 ^B,c^

### 3.8. Gene expression

**Fig 4:**
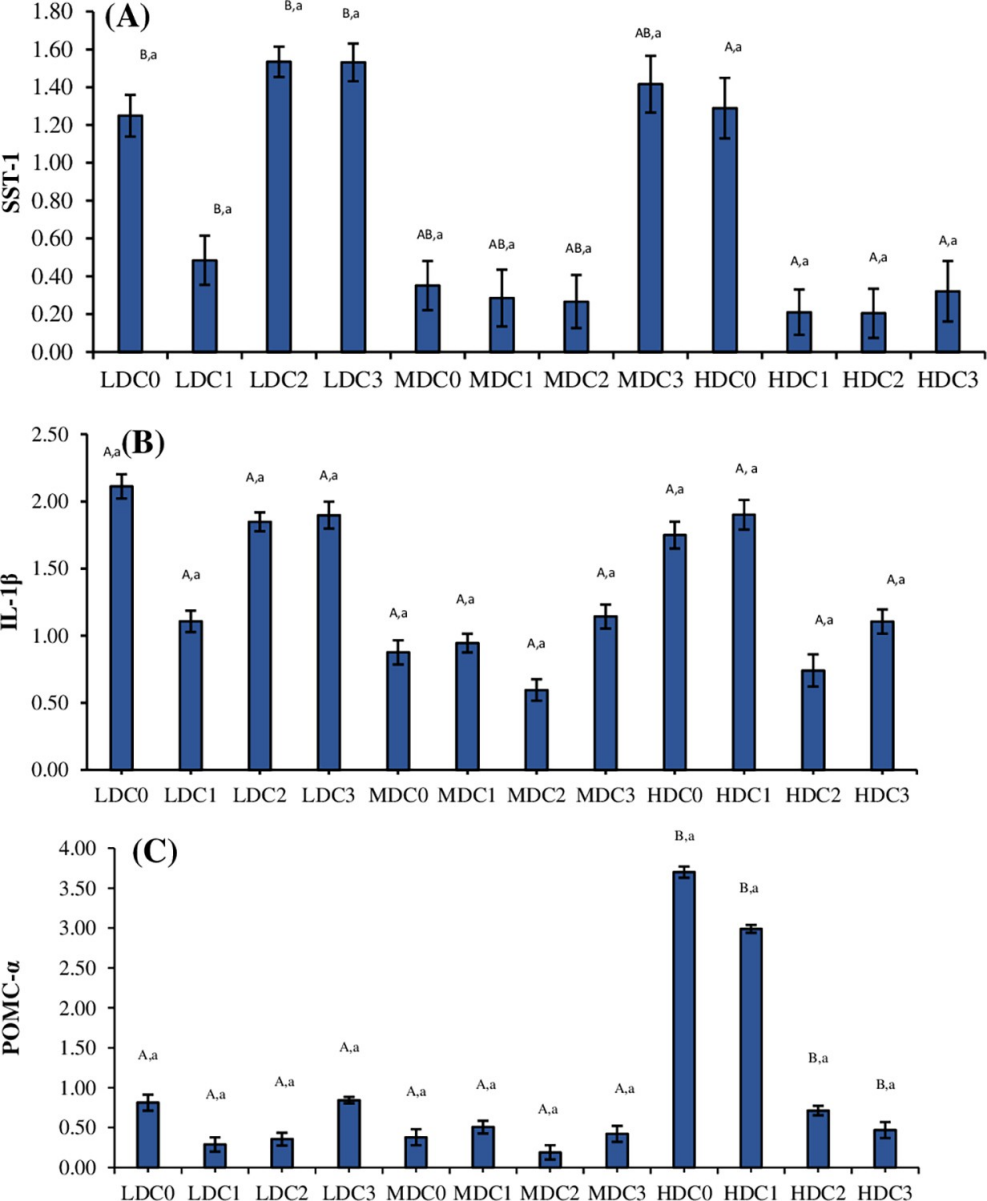
Levels of gene expression of (A) Somatostatin 1, (B) interleukin 1-β and (C) POMC-α (Mean ± SE) determined in three density treatment (LDC, MDC, HDC) having four curcumin supplementation levels (C0 = 0 mg/kg, C1 = 50 mg/kg, C2 = 100 mg/kg, C3 = 150 mg/kg). Superscripts represented as upper case letters show the comparison (P < 0.05) between three density treatments (LDC, MDC, HDC). Superscripts represented as lower-case letters show the comparison (P < 0.05) between four different levels of curcumin supplementation within one density treatment.

The expression of SST-1 gene (df_2_, F = 3.93, P = 0.03) and POMC-α (df_2_, F = 5.33, P = 0.008) was significantly different between three density treatment (Fig 4). However, the expression of interleukin 1-β (df_2_, F = 1.31, P = 0.28) was insignificantly different between three density treatment. Different levels of curcumin supplementation in three density (four in each density treatment) showed in significant effect on the levels of SST-1 (df_3_, F = 2.35, P = 0.08), interleukin 1-β (df_3_, F = 0.25, P = 0.86), and POMC-α (df_3_, F = 1.73, P = 0.17). Effect of density*curcumin calculated by Two-way ANOVA on the levels of SST-1 (df_6_, F = 1.65, P = 0.15), interleukin 1-β (df_6_, F = 0.36, P = 0.90) and POMC-α (df_6_, F = 1.54, P = 0.18) was insignificant.

### 3.9. Histological analysis

**Fig 5:**
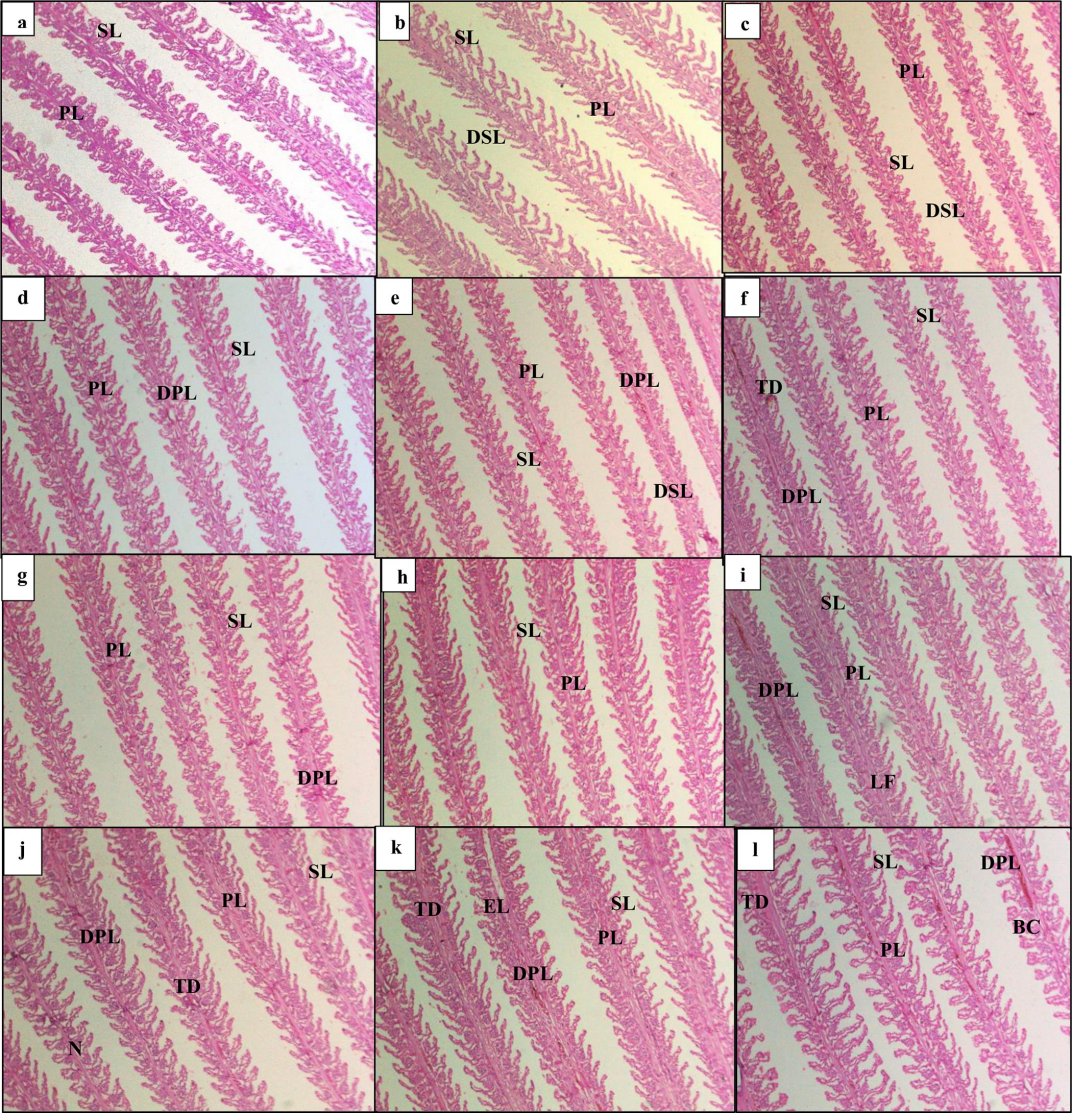
Histological changes in gills. Light micrographs of a paraffin section stained with eosin (10x). A(LDCO), B(LDC1), C(LDC2), D(LDC3), E(MDC0), F(MDC1), G(MDC2), H(MDC3), I(HDCO), J(HDC1), K(HDC2), L(HDC3). PL: Primary lamellae; SL: Secondary lamellae; DPL: Degeneration of primary lamellae; DSL: Degeneration of secondary lamellae; TD: Tissue debris; BC: Blood congestion; N: Necrosis; EL: Epithelial lifting; LF: Lamellar fusion.

Histology of gills was done for all treatments (density*curcumin) (Fig 5). Low density treatment showed minute disruption in structure of lamellae (Fig 5B–5D). Medium and high-density treatment showed high alteration in gills structure indicated by the degeneration of primary and secondary lamella (Fig 5E–5L), and tissue debris (Fig 5J–5l), compared with low density treatment. In high density treatment lamellar fusion (Fig 5I), necrosis (Fig 5J), epithelial lifting (Fig 5K) which is the detachment of epithelial cells from secondary lamellae and congestion in blood (Fig 5L) were observed. Low density treatment showed normal structure of gills including primary lamella and secondary lamella with less or no structural alterations.

## 4. Discussion

Studies have pointed out the adverse effect of overcrowding fish in high density conditions on their health and general wellbeing without any dietary supplementation [53,54]. The present research identified that dietary supplementation of curcumin showed a notable increase in growth parameters among treatments with different stocking densities. Specifically, treatment supplemented with dietary supplementation of curcumin (C1 = 50mg/kg) led to improved growth in fish raised under high-density conditions (HD = 4.50 kg/m^3^). Present study coincides with previous research conducted on various fish species in response to curcumin [31,35,55–60]. Growth performance of fish also linked with somatostatin gene-1 (SST-I). Present study noted high expression of SST-I gene in both low as well high stocking density treatments. This aligns with previous findings indicating that sst1 was upregulated in tilapia at high density [61] as well as in cichlids [62,63]. After the dietary supplementation of curcumin in current study at high density the expression of SST-1 drops which indicates the stimulatory effect in terms of growth.

Along with growth parameters, chemical composition, specifically crude protein slightly increased with dietary supplementation of curcumin within different stocking densities in current study. Increase in the chemical composition against curcumin supplementation has also been previously observed in Nile tilapia [64]. Present study aligns with previous findings indicating that SST-I was upregulated in tilapia at high density [61] as well as in cichlids [62,65]. This increase in the growth parameters and crude protein is due to digestive enhancer properties of curcumin [66,67]. Present study showed that the utilization of dietary supplementation of curcumin demonstrated a notable improvement in the activity of lipase, protease and amylase in the intestine as in previous studies [67].

In addition to digestive activities, dietary supplementation of curcumin improved the immune response of fish with different stocking densities [56]. The present study observed an elevated level of RBC and hemoglobin in fish raised at high density after dietary supplemented with curcumin compared to C0 treatment. Red blood cells (RBC) and hemoglobin are pivotal for transporting oxygen to fish tissues and eliminating harmful substances through the gills into the environment [68]. This finding is consistent with similar study on rainbow trout [56,57] and common carp [69]. The levels of triglycerides were observed lower due to improved lipid metabolism in fish, possibly by enhancing lipolysis and less fat accumulation in fish raised at high density due to dietary supplementation of curcumin compared to the C0 treatment [70,71]. Similar results have been observed in response to curcumin supplementation in rainbow trout [57], and amberjack (*Seriola dumerili*) where triglyceride and total cholesterol remained stable or even slightly decreased [72]. White blood cell (WBC) count, acting as a frontline defense in fish increases in response to infections [73]. This study noted an increase in WBC with dietary supplementation of curcumin at high density aligning with previous research on rainbow trout where curcumin supplementation enhanced WBC content [56,57]. The highest level of ALT and AST levels in high-density conditions were observed with C0 treatment which indicates potential harm to liver cells, as these enzymes are usually contained within cells but are released into the bloodstream when cell integrity is compromised [74]. Whereas these enzymes decreased in the treatments supplemented with dietary supplementation of curcumin as previously observed in common carp [75] and tilapia [76] as well.

The present study showed an increase in cortisol levels in fish with high-density treatment fed with C0 diet which showed stress level, changes in catecholamine levels, fluctuations in corticosteroid hormone levels and changes in blood profiles of fish [77] as compared to lower density groups aligning with previous study [15,78–80]. Whereases, curcumin supplemented treatments showed reduced levels of cortisol. This may be due to inhibitory effects of cortisol induced by ACTH and suppressed transcription of genes such as steroidogenic acute regulatory protein (StAR) and Cytochrome P450 (CYP)11a1 mRNAs in response to both ACTH and cAMP stimulation [81,82]. Current study aligns with previous studied conducted on dietary supplementation of curcumin in different fish species such as snakehead fish [83], tilapia [84], common carp [85] and Pacific white shrimp [86].

ACTH stops the activation of StAR and Cytochrome P450 (CYP) genes, as well as the pro-opiomelanocortin (POMC) gene. ACTH regulates cortisol through negative feedback control [87]. Present study indicated that treatment without curcumin supplementation (C0) at high stocking density showed markedly increased POMC-α expression due to higher stress level which triggers the activation of the hypothalamic-pituitary-adrenal (HPA) axis [88,89]. This process involves the release of corticotropin-releasing factor (CRF), which prompts the synthesis of pituitary pro-opiomelanocortin (POMC). POMC is then processed into adrenocorticotropic hormone (ACTH), leading to the stimulation of cortisol release via the melanocortin 2 receptor (MC2R) [90–92]. Whereases, the treatments supplemented with dietary curcumin exhibited decreased level of POMC-α gene expression compared to treatment C0 treatment suggests a positive outcome by reducing stress among high density treatment [93]. Addition of curcumin in diet significantly influences the expression of appetite-regulating neuropeptides like POMC in tilapia [94]. The present study aligns with previous findings conducted on tilapia [61].

The present research highlights an enhanced response of oxidative enzymes (CAT, SOD, GPx) under high-density conditions in C0 treatment. While, a decrease in these enzymes is observed with dietary curcumin supplementation, particularly at the C1 dose, especially in high-density situations. Curcumin has the ability to attach to free radicals and deliver a hydrogen atom, preventing anti-inflammatory, antibacterial, anticancer, antidiabetic, antiviral, antifungal agent and aiding in wound healing and immunomodulation [95–97] which plays a crucial role in its antioxidant effects [42,98]. A few studies have suggested that incorporating curcumin into the diet has positive effects on the performance and oxidative stability of other animal species i.e. chick [99] and quail [100] exposed to stressors other than high density such as chromium [101]; *Aeromonas salmonicida* [56]; *Aeromonas hydrophila* [34].

The production of oxidative enzymes including CAT, SOD and GPx alleviates adverse effects of high density after dietary curcumin supplementation compared to the C0 treatment. Similar results have been noted in various fish species [31,34,57,58,66,75,102,103]. MDA is also a marker of oxidative stress linked to lipid peroxidation [104], which increases in oxidative stress. However, contrary to existing literature, the study found that curcumin supplementation in diet did not increase levels of MDA [34,55,69].

Both antioxidant system and immune response are positively corelated. Raising fish, particularly in high-density environments that create elevated stress levels which can benefit from strengthening the fish immune system through the inclusion of natural antioxidants in their diet. Interleukin-1 beta (IL-1β), a proinflammatory cytokines released from activated macrophages plays a crucial role in regulating innate immune functions and inflammatory responses [105,106]. The current study suggests that curcumin supplement did not show any difference in IL-1β level in all treatments nor in different doses of dietary curcumin supplementation. Contrary to present study, other research has demonstrated that dietary curcumin reduced the mRNA levels of IL-1β *in vivo* and *in vitro* studies [107,108], including in snakehead [109], grass carp [31], tilapia [110], common carp [75,111], and Nile tilapia [64] and some studies also reported an increase in IL-1β levels in tilapia [112].

## 5. Conclusion

The Present study concluded that dietary supplementation of curcumin in different stocking density treatment showed better growth, antioxidant response and significant regulation of stress related genes (POMC-α). Curcumin proved to be an effective dietary supplement in alleviating oxidative stress, as evidenced by improvements in both antioxidant enzyme activity and the regulation of the POMC-α gene. While all curcumin doses were effective in mitigating various stress parameters, the C3 dose (150 mg/kg) yielded the most favorable outcomes, particularly in terms of enhanced antioxidant enzyme levels and suppressed POMC-α gene expression against high density = 4.50 kg/m^3^. This study highlights curcumin potential to enhance tilapia health against high stocking density. Dietary supplementation of curcumin C3 = 150mg/kg would be used in intensive farming against high stocking density to gain high yield without compromising fish well-being.

## Supporting information

S1 Table Output of two-way ANOVA indicating the effect of stocking density, curcumin and interactive effect of stocking density and curcumin on different parameters measured in the study. (XLSX)
